# Beyond the GRACE Score: A Multi-Biomarker Model for Improved Risk Stratification in Acute Coronary Syndromes

**DOI:** 10.3390/diagnostics16010012

**Published:** 2025-12-19

**Authors:** Gamze Yeter Arslan, Erkan Baysal

**Affiliations:** 1 Department of Cardiology, Kepez State Hospital, 07320 Antalya, Türkiye; 2 Department of Cardiology, Gazi Yaşargil Training and Research Hospital, 21000 Diyarbakır, Türkiye; dr.erkan.baysal@hotmail.com

**Keywords:** acute coronary syndrome, GRACE score, CAR, ALBI score, BUN/creatinine ratio

## Abstract

**Background:** The GRACE score is widely used to estimate early mortality in acute coronary syndromes (ACS), yet its ability to capture the complex interaction between inflammation, hepatic dysfunction, renal impairment, and myocardial injury remains limited. Integrating biomarkers that reflect these complementary physiological pathways may enhance risk prediction and allow earlier identification of high-risk patients. This study evaluated whether a multi-biomarker model incorporating the C-reactive protein/albumin ratio (CAR), the albumin–bilirubin (ALBI) score, and the blood urea nitrogen/creatinine (BUN/Cr) ratio provides incremental prognostic value beyond the GRACE score and traditional cardiac markers. **Methods:** This retrospective study included patients hospitalized with ACS. Baseline laboratory results were used to calculate CAR, ALBI, and BUN/Cr ratios. Troponin and hemoglobin values were recorded as standard cardiac and hematologic indicators. The primary outcome was in-hospital mortality. Logistic regression models, receiver operating characteristic (ROC) curve analysis, and comparisons of area under the curve (AUC) were performed to determine whether the multi-biomarker model improved risk stratification beyond the GRACE score alone. **Results:** Higher CAR, ALBI, and BUN/Cr values were each associated with increased in-hospital mortality. When combined with the GRACE score, the multi-biomarker model significantly improved predictive accuracy. The integrated model demonstrated a higher AUC compared with GRACE alone, indicating incremental prognostic value across inflammatory, hepatic, and renal pathways. **Conclusions:** A multi-biomarker strategy combining CAR, ALBI, and BUN/Cr ratios enhances early mortality prediction beyond the GRACE score in patients with ACS. Incorporating these readily available laboratory indices may help clinicians identify high-risk patients more precisely at the time of hospital admission.

## 1. Introduction

Coronary artery disease remains a leading cause of morbidity and mortality worldwide, and acute coronary syndromes (ACS) represent its most life-threatening clinical presentation [1]. Despite major advances in reperfusion strategies and guideline-directed medical therapy, early risk stratification at the time of hospital admission is still crucial to identify patients at high risk of adverse outcomes and to guide the intensity of monitoring and treatment [2]. The Global Registry of Acute Coronary Events (GRACE) risk score is among the most widely validated tools for predicting in-hospital and post-discharge mortality in ACS and is incorporated into international guidelines [3]. However, GRACE relies predominantly on clinical variables, hemodynamic parameters and standard laboratory data, and may not fully capture the complex interplay between systemic inflammation, hepatic congestion or dysfunction, renal impairment and myocardial injury that characterizes many ACS patients. The GRACE risk score remains the cornerstone of early risk stratification in acute coronary syndromes, growing evidence suggests that its predictive performance can be further refined by integrating biomarkers reflecting different pathophysiological axes. Contemporary studies have demonstrated that multimarker approaches incorporating inflammatory, metabolic, and organ-specific indices provide superior discrimination compared with clinical risk scores alone. Such strategies acknowledge that early mortality in ACS is driven not only by hemodynamic instability but also by systemic inflammatory activation, metabolic stress, and multi-organ dysfunction, which are not fully captured by GRACE variables alone [4,5,6].

Systemic inflammation plays a central role in plaque destabilization, microvascular dysfunction, and myocardial injury in acute coronary syndromes. C-reactive protein (CRP), a widely available acute-phase reactant synthesized by the liver, reflects inflammatory activation and has been associated with infarct size, left ventricular dysfunction, and mortality in ACS [7]. In contrast, serum albumin is a negative acute-phase reactant that reflects chronic inflammation, nutritional status, hepatic reserve, and overall disease burden, with low levels consistently linked to adverse outcomes in ACS and heart failure [8]. By integrating these opposing biological responses, the CRP/albumin ratio (CAR) provides a composite measure of systemic inflammatory stress and has been shown to predict both in-hospital and long-term mortality in patients with ACS and acute or chronic heart failure [9].

Beyond inflammation, hepatic dysfunction and congestion have emerged as important prognostic determinants in cardiovascular disease. The albumin–bilirubin (ALBI) score, originally developed for objective assessment of liver function using serum albumin and total bilirubin, has been increasingly applied in acute and chronic cardiac populations [10]. Prior studies have demonstrated that less negative ALBI values are independently associated with mortality and rehospitalization in heart failure and acute coronary syndromes, suggesting that hepatic involvement reflects not only impaired synthetic function but also systemic congestion and illness severity [11,12,13]. Given that ACS is frequently accompanied by right ventricular involvement, hypotension, and venous congestion, liver-related indices such as ALBI may represent a clinically relevant component of early risk stratification, although data in ACS remain limited.

Renal dysfunction represents another critical pathway linking systemic stress to adverse outcomes in ACS. Elevated blood urea nitrogen (BUN) and serum creatinine have long been associated with increased in-hospital and long-term mortality in acute myocardial infarction [14]. The BUN/creatinine ratio provides additional insight into renal hypoperfusion, neurohormonal activation, and catabolic stress beyond creatinine alone and has been proposed as a sensitive marker of prerenal azotemia and systemic illness. Emerging evidence indicates that an elevated BUN/creatinine ratio is associated with worse short-term outcomes in acute myocardial infarction and critically ill populations, even in the absence of advanced chronic kidney disease [15,16]. However, the incremental prognostic value of BUN/creatinine when evaluated alongside inflammatory and hepatic biomarkers has not been adequately explored in ACS cohorts.

Most prior studies have focused on single biomarkers or on one pathophysiologic axis at a time such as inflammation, hepatic function or renal function rather than on integrated multi-system assessment. CAR, ALBI and BUN/Cr each capture distinct but complementary aspects of the systemic response to ACS: inflammatory activity (CRP and albumin), hepatic reserve and congestion (albumin and bilirubin), and renal perfusion and catabolic stress (BUN and creatinine). At the same time, troponin remains the cornerstone biomarker of myocardial injury, and hemoglobin levels influence oxygen delivery and have been associated with outcomes in ACS. Combining these variables into a multi-biomarker model may therefore provide a more comprehensive view of patient risk than any single marker or clinical score alone.

The GRACE score has excellent discrimination for early mortality and remains the reference tool for risk stratification in ACS. Nevertheless, recent work has explored adding new electrocardiographic or biochemical markers to GRACE in order to refine risk prediction, suggesting that its performance can be further improved in selected populations [5]. To date, however, few studies have systematically examined whether the combined use of multi-system biomarkers simultaneously reflecting inflammatory, hepatic and renal pathways. provides incremental prognostic value beyond the GRACE score and conventional cardiac markers in patients with ACS.

In this context, we designed the present study to evaluate the prognostic impact of a multi-biomarker model incorporating the CRP/albumin ratio (CAR), the albumin–bilirubin (ALBI) score and the BUN/creatinine ratio, together with troponin and hemoglobin, in patients hospitalized with ACS. Our primary objective was to determine whether these readily available laboratory indices confer additional predictive value for in-hospital mortality beyond the GRACE risk score. We hypothesized that integrating inflammatory, hepatic and renal stress markers into a single model would enhance early risk stratification and allow a more precise identification of high-risk patients at the time of admission.

## 2. Methods

### 2.1. Study Design and Setting

This was a retrospective, observational cohort study conducted at two high-volume tertiary cardiology centers in Türkiye: Kepez State Hospital (Antalya) and Gazi Yaşargil Training and Research Hospital (Diyarbakır). All consecutive patients admitted with a diagnosis of acute coronary syndrome (ACS) between January 2022 and December 2024 were screened for eligibility. The study adhered to current reporting standards for observational studies and followed the principles of the Declaration of Helsinki.

### 2.2. Laboratory Measurements

All laboratory parameters were obtained from the institutional biochemistry and hematology laboratories using standardized, quality-controlled assays. For each patient, the following admission values were recorded:

- C-reactive protein (CRP, mg/L);
- Serum albumin (g/dL);
- Total bilirubin (mg/dL);
- Blood urea nitrogen (BUN, mg/dL);
- Serum creatinine (mg/dL);
- Total protein (g/dL);
- Hemoglobin (g/dL);
- High-sensitivity cardiac troponin (ng/L or equivalent assay-specific unit).

All laboratory parameters were obtained from the institutional biochemistry and hematology laboratories using standardized, quality-controlled assays. All measurements were based on the first available blood sample obtained at hospital admission to reflect the initial risk profile at presentation. If multiple blood samples were taken within the first 24 h, the earliest available measurement in the emergency department or coronary care unit was used. For the purpose of the present analysis, high-sensitivity cardiac troponin levels were defined as the first available measurement obtained at hospital admission, and serial or peak troponin values were not used.

### 2.3. Biomarker Calculations

#### 2.3.1. C-Reactive Protein/Albumin Ratio (CAR)

Inflammatory burden was represented by the C-reactive protein/albumin ratio (CAR), calculated as:

$$\mathrm{CAR}=\frac{\mathrm{CRP}\;(\mathrm{mg}/L)}{\mathrm{Albumin}\;(g/\mathrm{dL})}$$

Higher CAR values indicate a combination of increased inflammation and reduced negative acute-phase response.

#### 2.3.2. Albumin–Bilirubin Score (ALBI)

Hepatic function and congestion were assessed using the albumin–bilirubin score (ALBI), originally developed in hepatology. ALBI was calculated using the validated formula:

$$\mathrm{ALBI}=0.66\times {log}_{10}(\mathrm{bilirubin})-0.085\times \mathrm{albumin}$$

where total bilirubin is expressed in μmol/L and albumin in g/L. Unit conversions were performed when required (1 mg/dL bilirubin ≈ 17.1 μmol/L; albumin g/dL → g/L by multiplying by 10). Less negative (more positive) ALBI values reflect worse hepatic function and greater congestion.

### 2.4. BUN/Creatinine Ratio (BUN/Cr)

Renal stress and perfusion were evaluated with the BUN/creatinine ratio (BUN/Cr), calculated as:

$$\mathrm{BUN}/\mathrm{Cr}=\frac{\mathrm{BUN}\;(\mathrm{mg}/\mathrm{dL})}{\mathrm{Creatinine}\;(\mathrm{mg}/\mathrm{dL})}$$

Higher BUN/Cr values were interpreted as reflecting pre-renal azotemia, impaired renal perfusion and a higher catabolic state.

### 2.5. Conventional Biomarkers

Admission high-sensitivity troponin level was used as the principal marker of myocardial injury. Hemoglobin represented the oxygen-carrying capacity and overall hematologic status. All biomarker indices (CAR, ALBI, BUN/Cr, troponin, hemoglobin) were initially treated as continuous variables. In secondary analyses, patients were stratified into high- and low-risk groups using optimal cut-off values derived from receiver operating characteristic (ROC) curves.

### 2.6. GRACE Risk Score

For each patient, the in-hospital GRACE risk score was calculated using the standard algorithm, incorporating age, heart rate, systolic blood pressure, serum creatinine, Killip class, presence of cardiac arrest at admission, ST-segment deviation, and elevated cardiac biomarkers. GRACE was analyzed as a continuous variable and, in exploratory analyses, dichotomized using a conventional high-risk threshold. Patients with incomplete variables required for GRACE score calculation were excluded from the analysis, and no imputation methods were applied.

### 2.7. Outcomes

The primary endpoint was in-hospital all-cause mortality, defined as death from any cause during the index hospitalization. Secondary endpoints included development of cardiogenic shock, requirement for intravenous inotropic support, initiation of mechanical circulatory support (intra-aortic balloon pump or other devices), and occurrence of sustained ventricular tachycardia or ventricular fibrillation. Only events occurring during the index hospitalization were considered.

### 2.8. Statistical Analysis

Statistical analyses were performed using IBM SPSS Statistics version 26 (IBM Corp., Armonk, NY, USA). Data distribution for continuous variables was assessed with the Kolmogorov–Smirnov test and visual inspection of histograms and Q–Q plots. Normally distributed variables were reported as mean ± standard deviation (SD), and non-normally distributed variables as median with interquartile range (IQR). Categorical variables were summarized as counts and percentages.

Patients were divided into two groups according to in-hospital survival status (survivors vs. non-survivors). Baseline characteristics, GRACE scores, and biomarker levels were compared between groups using the independent samples *t*-test or Mann–Whitney U test for continuous variables and the chi-square or Fisher’s exact test for categorical variables.

Correlations between GRACE score and biomarker indices (CAR, ALBI, BUN/Cr, troponin, hemoglobin) were evaluated using Pearson or Spearman correlation coefficients as appropriate. Univariable logistic regression analyses were performed to identify potential predictors of in-hospital mortality, including age, sex, hypertension, diabetes mellitus, ACS subtype (STEMI vs. NSTE-ACS), Killip class, GRACE score, CAR, ALBI, BUN/Cr, troponin and hemoglobin. Variables with a *p* value < 0.10 in univariable analyses, together with clinically relevant factors, were entered into multivariable logistic regression models.

Because several key hemodynamic and laboratory variables, including Killip class, systolic blood pressure, heart rate, and renal function, are already incorporated into the GRACE score, additional adjustment for individual GRACE components was deliberately avoided to prevent overadjustment and multicollinearity, particularly given the limited number of in-hospital mortality events.

A base model including the GRACE score alone was constructed to predict in-hospital mortality. Biomarker indices (CAR, ALBI, BUN/Cr, troponin, hemoglobin) were then added stepwise and in combination to develop multi-biomarker models. Adjusted odds ratios (ORs) with 95% confidence intervals (CIs) were calculated. Model calibration was assessed using the Hosmer–Lemeshow goodness-of-fit test.

Discriminative ability was assessed using ROC curve analysis. The area under the ROC curve (AUC) and 95% CI were calculated for each model. AUCs for GRACE alone were compared with those of biomarker-augmented models using the DeLong method. Optimal cut-off values for CAR, ALBI and BUN/Cr were determined by maximizing the Youden index. A two-sided *p* value < 0.05 was considered statistically significant.

### 2.9. Ethics

The study was conducted in accordance with the Declaration of Helsinki. The protocol was reviewed and approved by the Gazi Yaşargil Training and Research Hospital Clinical Research Ethics Committee (Approval No. 743, dated 21 November 2025). Due to the retrospective design and use of anonymized data, the requirement for written informed consent was waived.

## 3. Results

During the study period, 468 patients were hospitalized with suspected ACS. After review of clinical records and final discharge diagnoses, 22 patients were excluded due to alternative diagnoses (e.g., myocarditis, pulmonary embolism, non-cardiac chest pain). The remaining 446 patients fulfilled ACS criteria based on ischemic symptoms, electrocardiographic ischemia, and elevated high-sensitivity cardiac troponin according to contemporary guideline definitions.

Additional exclusion criteria were then applied in a stepwise fashion:

- Age < 18 years (*n*= 4);
- Known congenital heart disease or significant valvular heart disease (moderate-to-severe stenosis or regurgitation) (*n* = 6);
- Permanent pacemaker or implantable cardioverter-defibrillator rhythm and complete left or right bundle branch block, precluding reliable ECG assessment (*n* = 9);
- Documented chronic inflammatory or autoimmune disease, active systemic infection, or known malignancy at admission (*n* = 14);
- End-stage liver disease, chronic dialysis-dependent kidney disease, or advanced chronic kidney disease stage 5 (*n* = 6);
- Cardiogenic shock requiring immediate mechanical circulatory support at presentation (*n* = 5);
- Missing key laboratory values (CRP, albumin, total bilirubin, BUN, creatinine, troponin or hemoglobin) or incomplete GRACE variables (*n* = 12).

**Figure 1:**
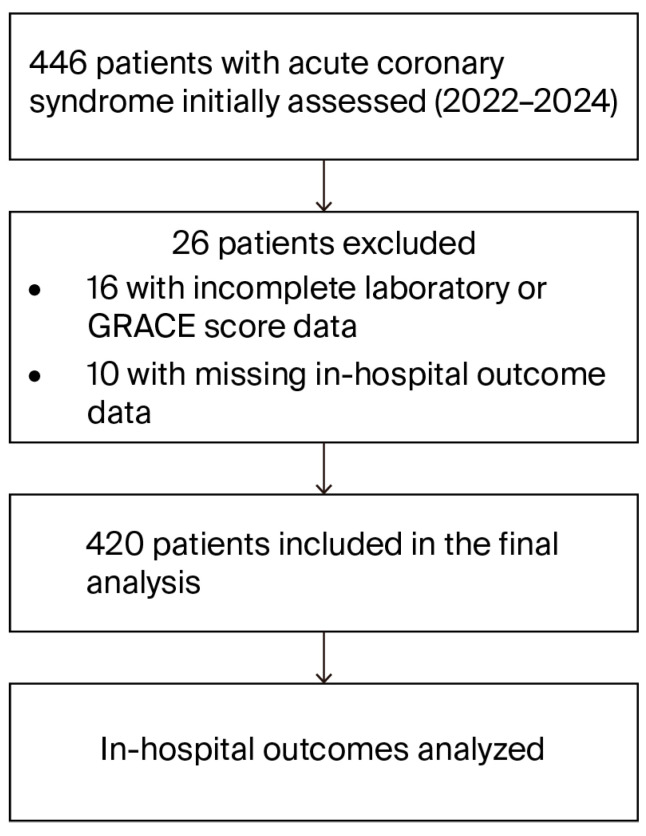
Patient Selection Flow Diagram. Legend: Flowchart illustrating patient screening, exclusions, and final cohort formation for the analysis of in-hospital outcomes in patients with acute coronary syndrome between 2022 and 2024. Patients with incomplete laboratory or GRACE score data or missing in-hospital outcome information were excluded from the final analysis.

After application of all criteria, a total of 420 patients with confirmed ACS and complete data were included in the final analysis. The patient selection process is depicted in Figure 1 (flow chart).

A total of 420 ACS patients were analyzed. The mean age was 63.4 ± 11.8 years, and 68.1% were male. STEMI presentation was observed in 54.3%, while 45.7% had NSTE-ACS. In-hospital mortality occurred in 34 patients (8.1%).

Baseline demographic, clinical, and laboratory characteristics according to survival status are presented in Table 1. Non-survivors were significantly older, had higher heart rates, lower systolic blood pressures, and substantially higher rates of Killip class II–IV. Serum creatinine and BUN levels were also significantly higher, whereas hemoglobin levels were lower in the mortality group. The GRACE risk score was markedly elevated among non-survivors compared with survivors (138 ± 24 vs. 119 ± 22; *p* < 0.001).

Biomarker distributions are shown in Table 2. CAR was more than twice as high in the mortality group. ALBI scores were significantly less negative (reflecting impaired hepatic function or congestion), and BUN/Creatinine ratios were substantially higher among non-survivors. Troponin values were dramatically elevated in the mortality group, while hemoglobin was significantly reduced.

**Figure 2:**
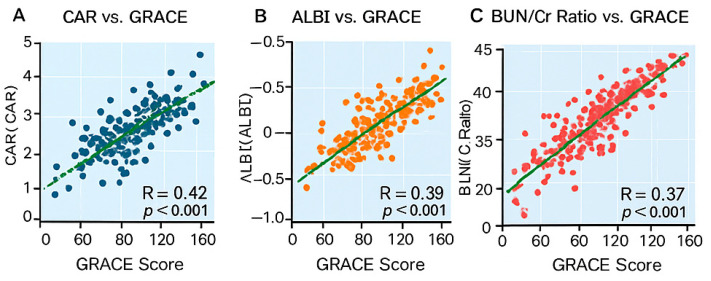
Correlation Plots. Legend: Figure 2 shows the correlations between GRACE score and three biomarkers: (**A**) CAR, (**B**) ALBI, and (**C**) BUN/creatinine ratio. All three indices demonstrated significant positive correlations with GRACE (*p* < 0.001), indicating that higher clinical risk in ACS is associated with increased inflammation, worse hepatic function, and impaired renal perfusion. Colored dots represent individual patients, with blue indicating CAR, orange indicating ALBI, and red indicating the BUN/creatinine ratio. The green line denotes the linear regression fit between the respective biomarker and the GRACE score.

Correlation analyses (Figure 2) demonstrated significant positive correlations between GRACE score and CAR (r = 0.44, *p* < 0.001), ALBI (r = 0.39, *p* < 0.001) and BUN/Creatinine (r = 0.47, *p* < 0.001). Troponin also correlated positively (r = 0.36, *p* < 0.001), whereas hemoglobin correlated negatively (r = −0.28, *p* < 0.001).

Univariable and multivariable logistic regression analyses are summarized in Table 3. After adjusting for GRACE score and clinically relevant covariates, CAR (OR 1.62), ALBI (OR 1.48), and BUN/Creatinine (OR 1.09) remained independent predictors of in-hospital mortality. Troponin was also independently associated with mortality (OR 1.31), whereas hemoglobin showed a protective effect (OR 0.78).

**Figure 3:**
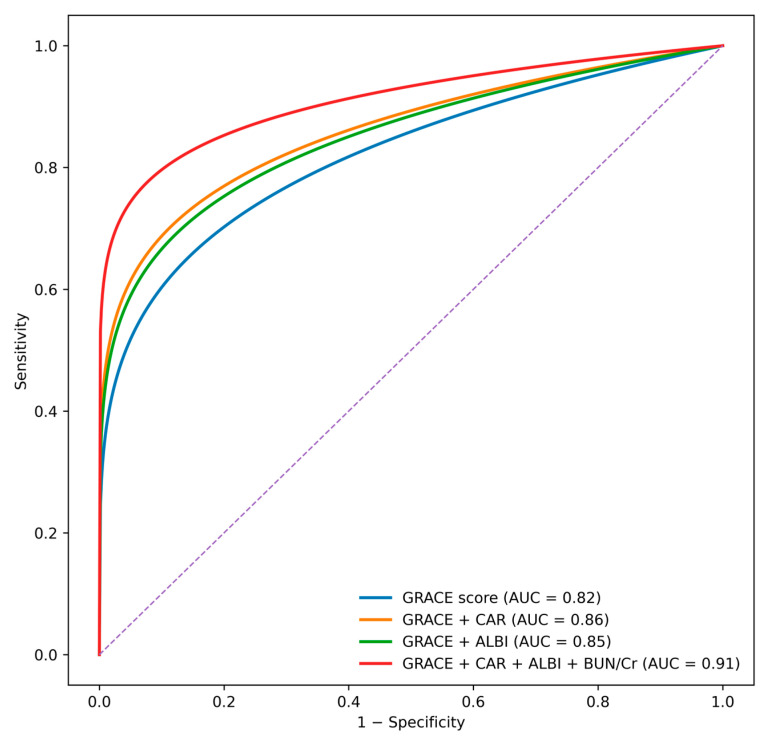
ROC curve comparison of the GRACE score alone and in combination with CAR, ALBI, and BUN/creatinine ratio for the prediction of in-hospital mortality. Area under the curve (AUC) values are shown for each model. The diagonal dashed line represents the reference line (AUC = 0.50). The addition of CAR, ALBI, and BUN/creatinine ratio to the GRACE score improved overall model discrimination.

ROC curve comparisons (Figure 3) revealed that the GRACE score alone had an AUC of 0.82. The addition of individual biomarkers improved discrimination, with AUC values of 0.86 for CAR, 0.85 for ALBI, and 0.88 for the BUN/creatinine ratio. The complete multi-biomarker model incorporating CAR, ALBI, and BUN/creatinine ratio achieved the highest discriminative performance (AUC 0.91) and significantly outperformed the GRACE score alone according to DeLong test comparisons (*p* < 0.001).

Cut-off values determined by Youden index were:

- CAR > 2.8;
- ALBI > −2.60;
- BUN/Creatinine > 20.5.

These thresholds were strongly associated with increased in-hospital mortality.

## 4. Discussion

In this retrospective cohort of 420 patients with acute coronary syndrome (ACS), we found that three readily obtainable multi-system indices CAR, ALBI, and the BUN/creatinine ratio were strongly and independently associated with in-hospital mortality, even after adjustment for the established GRACE risk score. The addition of these biomarkers meaningfully improved the prognostic performance of GRACE, with the full model incorporating CAR, ALBI, BUN/Cr, troponin, and hemoglobin achieving the highest discriminative accuracy. These findings highlight the complex and systemic nature of ACS pathophysiology, in which mortality is influenced not only by the extent of myocardial injury but also by the degree of inflammatory activation, hepatic congestion, and renal perfusion abnormalities.

Previous studies have firmly established the prognostic importance of systemic inflammation in ACS. Elevated CRP levels have consistently been linked to increased mortality and adverse cardiovascular events, while lower albumin levels indicate poor nutritional and inflammatory status, endothelial dysfunction, and heightened oxidative stress [17]. CAR merges these two biologically relevant pathways into a single composite index. Although emerging evidence suggests that CAR may predict long-term outcomes in STEMI patients, most prior analyses either lacked adjustment for validated clinical risk scores or did not compare CAR with other organ-based indices [18]. Our findings extend the existing literature by demonstrating that CAR remains an independent predictor of in-hospital mortality even after robust adjustment for GRACE, and by quantifying its incremental prognostic value. This reinforces the notion that early inflammatory activation plays a pivotal role in short-term mortality among ACS patients.

The ALBI score, originally developed to assess liver function and synthetic reserve in patients with chronic liver disease, has recently attracted attention in cardiovascular research. Several studies in heart failure populations have suggested that ALBI reflects hepatic congestion resulting from elevated right-sided pressures and systemic venous hypertension [19]. Limited evidence exists regarding ALBI in ACS, and most available studies have been confined to small STEMI cohorts undergoing primary PCI [20]. These earlier works showed that ALBI was associated with higher in-hospital mortality, but were restricted by small sample sizes, lack of multi-marker comparison, and absence of adjustment for GRACE. Our results not only validate ALBI as an independent predictor of mortality in a larger and more heterogeneous ACS population, but also demonstrate that it significantly improves risk discrimination when added to GRACE. This suggests that hepatic congestion and reduced hepatic synthetic capacity represent clinically meaningful contributors to early ACS prognosis.

ALBI score was originally developed as an objective measure of hepatic function; however, in the context of acute coronary syndromes, its prognostic value likely extends beyond isolated hepatic congestion. Because echocardiographic parameters reflecting right ventricular function or venous congestion were not available in the present study, ALBI should be interpreted cautiously as a surrogate marker of overall systemic illness severity rather than a direct indicator of hepatic congestion alone. This interpretation is consistent with the concept that acute coronary syndromes represent a multisystem stress condition, in which hepatic dysfunction may reflect the cumulative effects of inflammation, hemodynamic compromise, and neurohormonal activation.

Renal dysfunction is another well-recognized determinant of adverse outcomes in ACS. While creatinine is included in GRACE and other risk scores, the BUN/creatinine ratio provides additional information regarding renal perfusion, neurohormonal activation, and catabolic stress. Prior studies in heart failure populations have found that BUN/Cr outperforms creatinine alone in predicting mortality, but its role in ACS remains underexplored [21]. A few small studies have reported associations between BUN/Cr and short-term outcomes in STEMI, yet none have integrated this marker into a comprehensive multi-organ prognostic framework [22]. Our study demonstrates that BUN/Cr is not only strongly associated with in-hospital mortality in ACS, but also independently predicts mortality after adjustment for GRACE. Moreover, the substantial increase in AUC when BUN/Cr is added to GRACE underscores its value as a simple, inexpensive, and informative renal perfusion marker that may be particularly useful in emergency settings.

Traditional cardiac biomarkers such as troponin have long been recognized as essential tools for risk stratification, and the prognostic impact of baseline hemoglobin is increasingly acknowledged, with anemia contributing to increased myocardial oxygen demand and worsened ischemia. Baseline hemoglobin reflects both oxygen-carrying capacity and overall physiological reserve. Anemia may exacerbate myocardial ischemia by impairing oxygen delivery and increasing cardiac workload, thereby amplifying the adverse effects of systemic inflammation and organ dysfunction. This pathophysiological interplay likely explains the independent association between lower hemoglobin levels and in-hospital mortality observed in our cohort, consistent with prior large-scale ACS studies [23,24]. Although these markers are widely studied, most prior investigations have evaluated them individually rather than in combination with multi-organ indices reflecting inflammatory, hepatic, and renal dysfunction. By integrating troponin and hemoglobin into a model with CAR, ALBI, and BUN/Cr, our study provides a more comprehensive assessment of physiological stress across multiple organ systems during ACS.

Acute glucometabolic dysregulation has been increasingly recognized as an important prognostic factor in acute coronary syndromes. Admission hyperglycemia and stress-related disturbances in glucose metabolism have been shown to independently predict adverse in-hospital and long-term outcomes in both diabetic and non-diabetic ACS populations. These findings suggest that glucometabolic markers reflect systemic stress, inflammatory activation, and microvascular vulnerability that may not be fully captured by conventional clinical risk scores. In this context, glucometabolic parameters may represent a complementary dimension to biomarker-based risk stratification models focused on inflammation, hepatic function, and renal impairment [25].

One of the most notable contributions of this study is its demonstration that a combined multi-system model substantially enhances mortality prediction beyond the GRACE score alone. While GRACE is widely used and validated, its development predates the widespread adoption of newer multi-organ indices. Our results indicate that incorporating biomarkers reflective of inflammatory burden, hepatic function, and renal perfusion can modernize and augment the prognostic performance of GRACE. The observed improvement in AUC from 0.82 (GRACE alone) to 0.91 (full model) represents a clinically meaningful enhancement in risk stratification, with potential implications for triage, treatment decisions, and monitoring intensity in the early hours of ACS care. Although individual clinical variables such as systolic blood pressure, heart rate, Killip class, renal function, and hemoglobin are clinically relevant, many of these parameters are already incorporated within the GRACE score. Therefore, GRACE-based adjustment was deliberately preferred to preserve model parsimony and to avoid overadjustment and multicollinearity, particularly given the limited number of in-hospital mortality events.

Our findings support the growing concept that acute coronary syndromes should be viewed as a systemic, multi-organ stress condition rather than an isolated myocardial event. Multimarker models integrating inflammatory, hepatic, renal, and hematologic indices have been shown to outperform single-marker approaches by capturing complementary pathophysiological mechanisms. In line with previous multimarker studies, the substantial improvement in discriminative performance observed in our analysis suggests that combining CAR, ALBI, and BUN/creatinine with the GRACE score provides a more holistic assessment of early mortality risk [26]. Recent studies have further emphasized the prognostic relevance of systemic inflammatory indices, such as the systemic immune-inflammation index and related composite markers, in predicting in-hospital and short-term mortality among patients with acute coronary syndromes, supporting the concept that integrated inflammatory scores capture prognostic information beyond traditional clinical risk models [27]. In parallel, accumulating evidence indicates that hepatic function–based scores, including the albumin–bilirubin (ALBI) score, are associated with adverse cardiovascular outcomes and mortality in acute cardiac settings, reinforcing the role of hepatic involvement as a marker of global illness severity [28].

Compared with previous biomarker studies, the strengths of our research include a relatively large and heterogeneous cohort, inclusion of both STEMI and NSTE-ACS patients, comprehensive adjustment for GRACE, and simultaneous evaluation of five distinct physiologic domains. Most earlier ACS biomarker studies were limited by small sample sizes, single marker analyses, lack of adjustment for clinical risk scores, or homogeneous populations. Our study addresses these gaps by providing a robust and multidimensional prognostic framework that reflects the systemic pathophysiology of ACS.

These findings collectively emphasize that ACS is best conceptualized as a condition of both myocardial and extra-cardiac stress. Inflammatory activation, hepatic congestion, and impaired renal perfusion appear to interact synergistically, leading to higher mortality risk. Early identification of patients exhibiting multi-system dysfunction may therefore support more intensive hemodynamic monitoring, earlier invasive evaluation, and prioritization for aggressive therapeutic strategies.

## 5. Limitations

This study has several limitations. First, its retrospective observational design inherently introduces potential selection and information biases, even though consecutive patient inclusion was used to mitigate these factors. Second, although the sample size is larger than most single-center biomarker studies, the data were derived from two centers within the same national healthcare system, which may limit external generalizability. Third, we evaluated only in-hospital mortality, and therefore could not assess the impact of these biomarkers on long-term outcomes. Fourth, biomarker dynamics—such as changes in CAR, ALBI, or BUN/Cr during hospitalization—were not evaluated; serial measurements might yield additional prognostic insights. Fifth, echocardiographic parameters, including left ventricular ejection fraction and right ventricular function, were not consistently available and therefore not incorporated into the models.

Although secondary clinical events such as cardiogenic shock and ventricular arrhythmias were recorded during hospitalization, these outcomes were not systematically adjudicated and were not included in the final analyses. Therefore, in-hospital mortality was selected as the primary endpoint of the study. In addition, the relatively limited number of mortality events (*n* = 34) may have reduced the statistical power of multivariable models and should be considered when interpreting the results.

The present study focused exclusively on in-hospital mortality, an endpoint primarily driven by acute hemodynamic instability and early systemic stress. Although CAR, ALBI, and BUN/creatinine ratio have demonstrated prognostic relevance for long-term cardiovascular outcomes in prior studies, the retrospective design and short-term follow-up of the current analysis do not allow assessment of long-term risk. Therefore, the proposed biomarker-based model should be interpreted as an early risk stratification tool rather than a predictor of long-term outcomes, which limits the generalizability of our findings beyond the in-hospital period.

Given the limited number of in-hospital mortality events and the risk of model overfitting, net reclassification improvement and decision curve analyses were not performed, as these approaches require a larger number of outcome events for reliable estimation.

Finally, although GRACE score was included, unmeasured confounders such as frailty, nutritional status, or unrecorded comorbidities may still influence outcomes.

## 6. Conclusions

In this study of 420 patients with acute coronary syndrome, we demonstrated that CAR, ALBI, and the BUN/creatinine ratio are independent, powerful predictors of in-hospital mortality. When added to the GRACE score, each biomarker significantly improved risk discrimination, and the full multi-biomarker model achieved the highest predictive accuracy. These findings underscore the clinical importance of recognizing ACS as a multi-organ stress condition and support the incorporation of inflammatory, hepatic, renal, cardiac, and hematologic markers into early risk stratification. Future prospective studies are warranted to validate these findings and determine whether biomarker-guided risk assessment can enhance clinical decision-making and outcomes.

## Figures and Tables

**Table 1:** Baseline Clinical and Laboratory Characteristics of Survivors and Non-survivors.

Variable	Survivors (*n* = 386)	Non-Survivors (*n* = 34)	*p* Value
Age, years	62.8 ± 11.7	69.2 ± 10.3	0.001
Male sex, %	67.4%	76.5%	0.28
STEMI, %	53.0%	67.6%	0.04
Killip class II–IV, %	19.7%	61.8%	<0.001
Systolic BP, mmHg	128 ± 23	112 ± 25	<0.001
Heart rate, bpm	79 ± 15	88 ± 18	0.002
Creatinine, mg/dL	0.98 ± 0.26	1.21 ± 0.34	<0.001
BUN, mg/dL	17.5 ± 6.1	24.3 ± 7.5	<0.001
Hemoglobin, g/dL	13.2 ± 1.6	11.8 ± 1.4	<0.001
GRACE score	119 ± 22	138 ± 24	<0.001

Legend: Baseline demographic, clinical, hemodynamic, and laboratory characteristics according to in-hospital mortality. Continuous variables are presented as mean ± standard deviation, and categorical variables are presented as percentages. Group comparisons were performed using appropriate parametric or non-parametric tests according to data distribution.

**Table 2:** Distribution of Biomarker Indices in Survivors and Non-survivors.

Biomarker	Survivors (*n* = 386)	Non-Survivors (*n* = 34)	*p* Value
CAR	1.42 (0.80–2.30)	3.48 (2.10–5.60)	<0.001
ALBI score	−2.78 ± 0.37	−2.45 ± 0.42	<0.001
BUN/Cr ratio	17.9 ± 5.8	24.6 ± 7.1	<0.001
Troponin (ng/L)	2540 (1200–6800)	7680 (3100–15,400)	<0.001
Hemoglobin (g/dL)	13.2 ± 1.6	11.8 ± 1.4	<0.001

Legend: Distribution of biomarker indices according to in-hospital mortality. Biomarkers with non-normal distribution (CAR and troponin) are presented as median (interquartile range), whereas normally distributed variables (ALBI score, BUN/creatinine ratio, and hemoglobin) are presented as mean ± standard deviation. Group comparisons were performed using appropriate parametric or non-parametric tests.

**Table 3:** Univariable and Multivariable Logistic Regression Analysis for Predictors of In-hospital Mortality.

Variable	Univariable OR (95% CI)	*p* Value	Multivariable OR (95% CI)	*p* Value
GRACE score	1.04 (1.02–1.06)	<0.001	1.02 (1.00–1.04)	0.03
CAR	1.88 (1.45–2.45)	<0.001	1.62 (1.21–2.18)	0.001
ALBI	1.21 (1.10–1.34)	<0.001	1.48 (1.11–1.96)	0.007
BUN/Cr	1.12 (1.07–1.18)	<0.001	1.09 (1.04–1.15)	<0.001
Troponin	1.46 (1.19–1.81)	<0.001	1.31 (1.09–1.57)	0.003
Hemoglobin	0.69 (0.58–0.84)	<0.001	0.78 (0.64–0.95)	0.014

Legend: Univariable and multivariable logistic regression analyses were performed to identify predictors of in-hospital mortality. The multivariable model was adjusted for the GRACE score. Model discrimination improved from an AUC of 0.82 for the GRACE score alone to 0.91 with the addition of CAR, ALBI, and BUN/creatinine ratio. Model calibration was assessed using the Hosmer–Lemeshow goodness-of-fit test (*p* > 0.05).

## Data Availability

The raw data supporting the conclusions of this article will be made available by the authors on request.
